# Supplementary material for: Generation of a zebrafish neurofibromatosis model via inducible knockout of nf2
Source: bioRxiv. 2024 Apr 27:2024.04.23.590787. Preprint. [Version 1] doi: 10.1101/2024.04.23.590787 (PMC11071375; doi:10.1101/2024.04.23.590787)
Supplement: Supplement 1 [file NIHPP2024.04.23.590787v1-supplement-1.pdf]

bioRxiv preprint doi: https://doi.org/10.1101/2024.04.23.590787; this version posted April 27, 2024. The copyright holder for this preprint (which was not certified by peer review) is the author/funder, who has granted bioRxiv a license to display the preprint in perpetuity. It is made available under aCC-BY-NC-ND 4.0 International license.

Supplementary Table 1

Reagent or Resource	Source, Identifier
**Experimental Models: Organisms/Strains**	
ABWT	ZIRC, ZDB-GENO-960809-7
Tg(-7.2sox10:mRFP)	ZDB-TGCONSTRCT-080321-2
Tg(pU6x:*nf2*-4sgRNA)	This paper
Tg(HOTCre:Cas9)	ZDB-TGCONSTRCT-170418-5
**Plasmids**	
pCS2-Cre.zf1	Addgene; Plasmid #61391
pGGDestTol2LC-*nf2*-4sgRNA	This paper; modified from addgene plasmid Plasmid #64242
pU6a-*nf2a*-gRNA1	This paper; modified from addgene plasmid Plasmid #64245
pU6a-*nf2a*-gRNA3	This paper; modified from addgene plasmid Plasmid #64246
pU6b-*nf2b*-gRNA1	This paper; modified from addgene plasmid Plasmid #64247
pU6c-*nf2b*-gRNA2	This paper; modified from addgene plasmid Plasmid #642428
**Hybridization chain reaction (HCR) probes**	
sox10 HCR probes	This paper; Molecular Technologies

bioRxiv preprint doi: https://doi.org/10.1101/2024.04.23.590787; this version posted April 27, 2024. The copyright holder for this preprint (which was not certified by peer review) is the author/funder, who has granted bioRxiv a license to display the preprint in perpetuity. It is made available under aCC-BY-NC-ND 4.0 International license.

foxc1b HCR probes	This paper; Molecular Technologies
nf2a HCR probes	This paper; Molecular Technologies
nf2b HCR probes	This paper; Molecular Technologies
igfbp2a HCR probes	This paper; Molecular Technologies
**Antibodies**	
NF2/Merlin antibody	GENETEX - Cat No GTX48502
Histone H3 antibody	Abcam - ab1791
phospho-histone3 (pH3) antibody	Abcam - Cat No ab14955
**Oligonucleotides**	
**PCR primers**	
*nf2a*_gRNA1 (w/o PAM) - GGATCATGATGTGCCCAAAG	This paper; IDT
*nf2a*_gRNA3 (w/o PAM) - GTACGACGTCAAAGACACTG	This paper; IDT
*nf2b*_gRNA1 (w/o PAM) - GGAGGAGAAGATAACCGCT	This paper; IDT
*nf2b*_gRNA2 (w/o PAM) - GGCCAATGGTCCGGCATACC	This paper; IDT

bioRxiv preprint doi: https://doi.org/10.1101/2024.04.23.590787; this version posted April 27, 2024. The copyright holder for this preprint (which was not certified by peer review) is the author/funder, who has granted bioRxiv a license to display the preprint in perpetuity. It is made available under aCC-BY-NC-ND 4.0 International license.

nf2a gR3_exonprimer RP - TTCTTGTCCATTTTCAGCCAGG	This paper; IDT
nf2b gR1_exonprimer FP - TGATGCAGTATCAGATGACACCAG	This paper; IDT
nf2b gR1_exonprimer RP - TTTCTGTGTTCAGCATACCAAGC	This paper; IDT
nf2b gR2_exonprimer FP - TGGAAAGGGAAAGATTTATTTGACC	This paper; IDT
nf2b gR2_exonprimer RP - CAGGCTTCAGCCAGGCAT	This paper; IDT
beta-actin FP - CGAGCTGTCTTCCCATCCA	Tang R et al, Acta Biochimica et Biophysica Sinica,2007; IDT
beta-actin RP - TCACCAACGTAGCTGTCTTTCTG	Tang R et al, Acta Biochimica et Biophysica Sinica,2007; IDT
**T7 endonuclease assay PCR primers**	
nf2b_gR1_FP - CGGGGACTACGATCCAAACTT	This paper; IDT
nf2b_gR1_RP - ACATGTCTAGATCCTGGGCTAT	This paper; IDT
nf2b_gR2&3_FP -	This paper; IDT

bioRxiv preprint doi: https://doi.org/10.1101/2024.04.23.590787; this version posted April 27, 2024. The copyright holder for this preprint (which was not certified by peer review) is the author/funder, who has granted bioRxiv a license to display the preprint in perpetuity. It is made available under aCC-BY-NC-ND 4.0 International license.

GGGTCTGAAAAAGAAGCAACCAAA	
nf2b_gR2&3_RP - ATCAGGCTTCAGCCAGGCATA	This paper; IDT
nf2a_gR1_FP - GCAGAGTGCAACACTTATAATGACT	This paper; IDT
nf2a_gR1_RP - GAAACCGGCCCCATGAGTAA	This paper; IDT
nf2a_gR2&3_FP - GTGGCGAGGAAAGGATCTGT	This paper; IDT
nf2a_gR2&3_RP - TTCTTGTCCATTTTCAGCCAGG	This paper; IDT

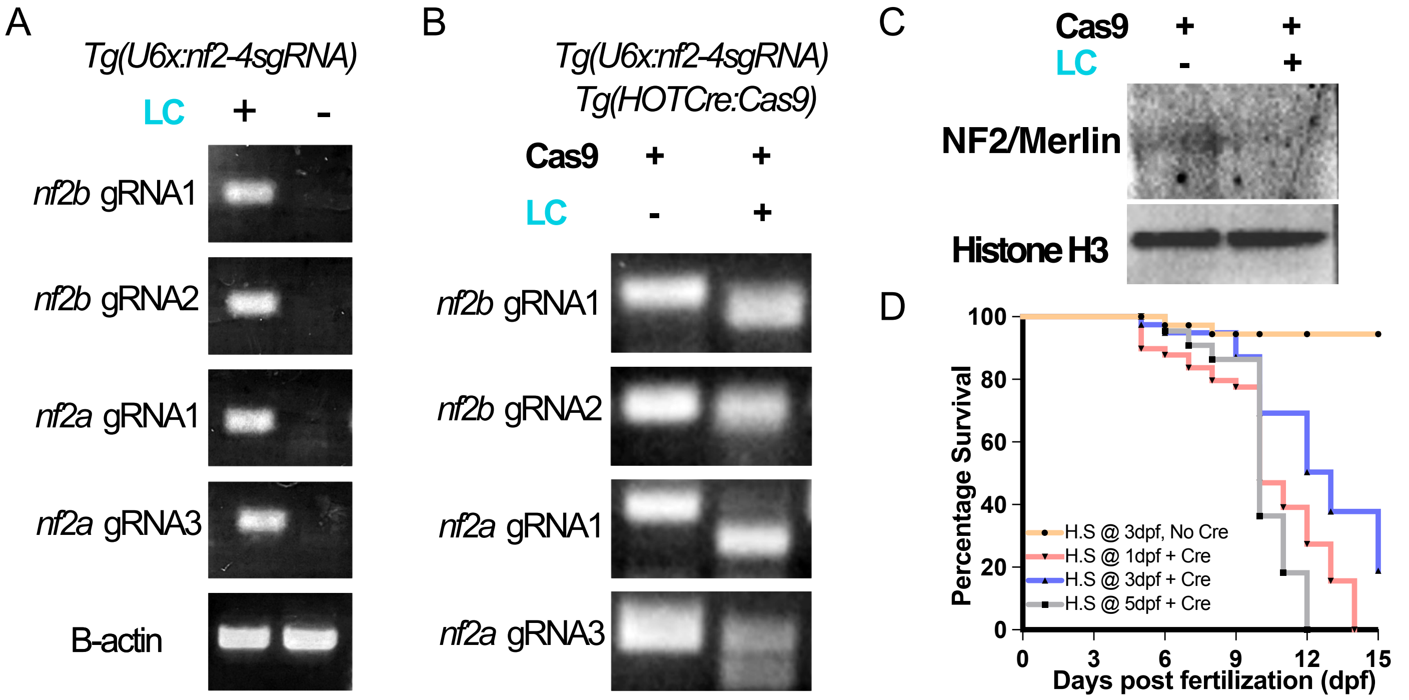

Suppl Fig 1: Validation of stable transgenic line *Tg(U6x:nf2-4sgRNA)::HOTCre:Cas9.* (A) Agarose gel images showing the expression of the guide RNAs in the stable transgenic line. (B) Agarose gel image of T7 endonuclease assay for *nf2* guide RNA target regions. (C) Western blot image showing down regulation of NF2 protein in *nf2* knockouts, Histone H3 was used as the loading control. (D) Survival plot of *Tg(U6x:nf2-4sgRNA):HOTCre:Cas9* after heatshock in the presence/absence of Cre-recombinase mRNA at different embryonic/larval stages. LC + indicates lens cerulean positive (contains *nf2* targeting guide RNAs) and Cas9 + indicates cardiac GFP positive (expresses Cas9 upon heatshock) embryos were used for the experiment.

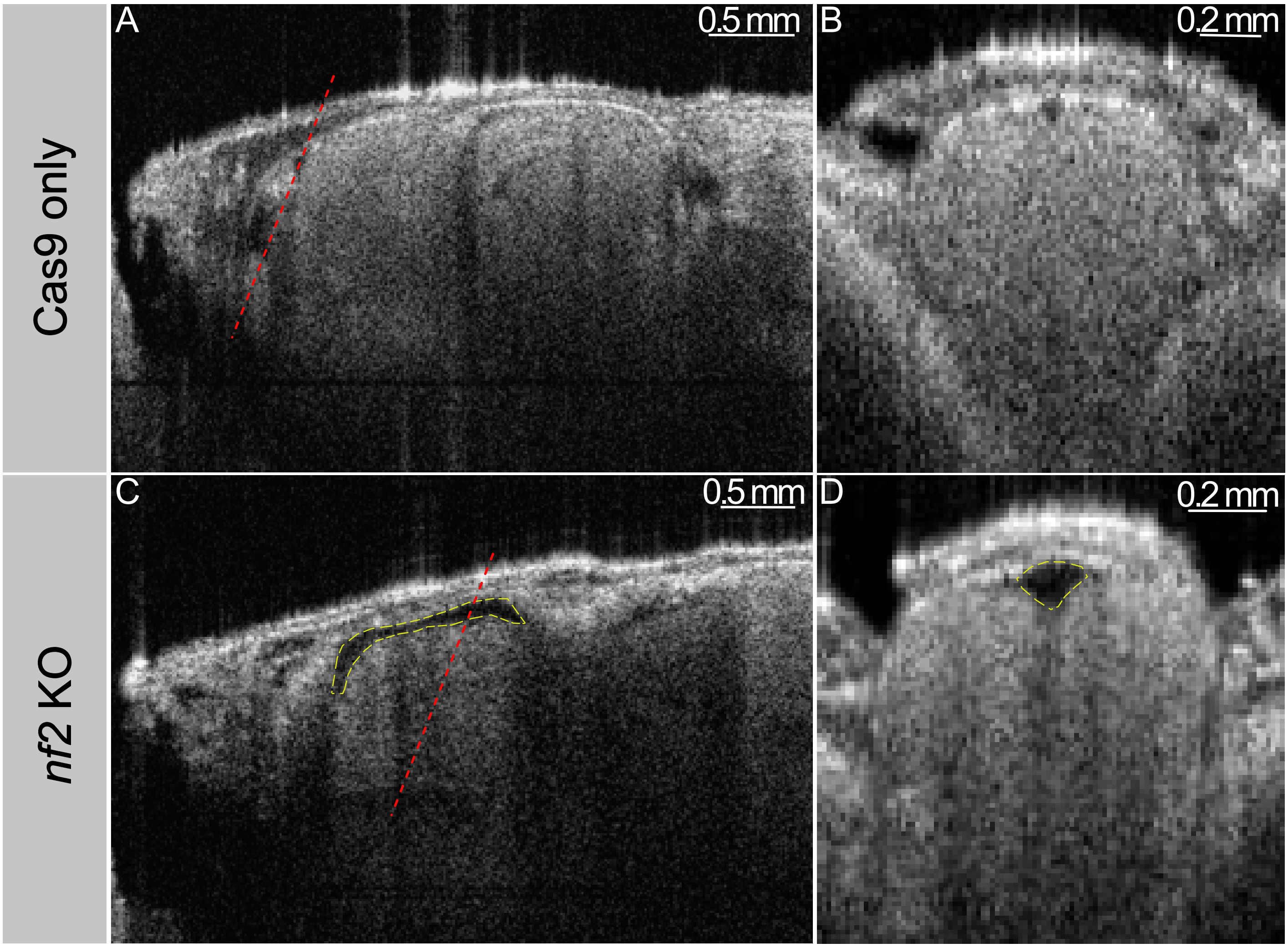

Suppl Fig 2: Nf2 knockout adult zebrafish display enlarged telencephalic ventricles. OCT images of euthanized adult zebrafish revealed enlarged telencephalic ventricles (highlighted with dashed yellow line) in *nf2* knockout (C,D) animals as compared to Cas9-only controls (A-B). Transverse section taken as indicated by red dashed line.
